# Muscle Synergy During Cutting Movements in Athletes with a History of Groin Pain

**DOI:** 10.3390/sports13100338

**Published:** 2025-10-02

**Authors:** Hiromi Saito, Nadaka Hakariya, Teerapat Laddawong, Toshiaki Soga, Tatsuya Moteki, Koji Kaneoka, Naoto Matsunaga, Norikazu Hirose

**Affiliations:** 1 Graduate School of Sport Sciences, Waseda University, Saitama 169-8050, Japan; hiromisaito.physio@gmail.com (H.S.);; 2 Department of Life Sciences, Graduate School of Arts and Sciences, The University of Tokyo, Tokyo 113-0033, Japan; 3 Department of Rehabilitation for the Movement Functions, Research Institute of National Rehabilitation Center for the Disabled, Saitama 359-8555, Japan; 4 Department of Physical Therapy, Faculty of Allied Health Science, Thammasat University, Pathum Thani 12121, Thailand; 5 Graduate School of Engineering and Science, Shibaura Institute of Technology, Saitama 337-8570, Japan; 6 Research Fellow of Japan Society for the Promotion of Science, Tokyo 102-0083, Japan; 7 Faculty of Sport Sciences, Waseda University, Saitama 169-8050, Japan; 8 General Education Core Curriculum Division, Seigakuin University, Saitama 362-8585, Japan

**Keywords:** groin injury, muscle coordination, EMG, NMF

## Abstract

This study examined muscle coordination during cutting movements in athletes with a history of groin pain. A total of 15 athletes who had experienced groin pain in the past two years (GP) and 14 healthy controls (CON) participated. Electromyography (EMG) and ground reaction force (GRF) data were collected, and EMG was analyzed using non-negative matrix factorization to extract muscle synergies. Three synergies were identified in both groups: Synergy 1 (landing), Synergy 2 (deceleration), and Synergy 3 (acceleration). No group differences were observed in GRF. However, compared with the CON, the GP demonstrated a 58.1% greater contribution of the latissimus dorsi and a 31.5% greater contribution of the erector spinae (SES) in Synergy 1, suggesting excessive trunk involvement during landing. In Synergy 2, SES contribution was 97.0% lower in the GP. In Synergy 3, the external oblique contribution decreased by 118.4%, while rectus abdominis contribution increased by 54.3%. These muscles are critical for pelvic stability, and their altered contributions indicate disrupted neuromuscular coordination in athletes with GP.

## 1. Introduction

### 1.1. Background of Groin Pain

Groin pain syndrome is common in athletes, such as football [1] and rugby players [2], who perform repetitive cutting movement [1]. History of groin pain has been reported as a risk factor for groin pain [3]. In 29% of individuals with a history of adductor-type groin pain experience recurrence [4], understanding the physical characteristics of these individuals is important for preventing re-injury.

### 1.2. Muscle Activity and Ground Reaction Force (GRF) Characteristics of Groin Pain

Previous research on groin pain has identified dysfunctions in both the trunk [5] and lower limbs [6], such as reduced the internal oblique and hip adductor muscles activation [5], using electromyography (EMG). Cowan et al. reported dysfunction of the internal oblique and transverse abdominis muscles in patients with groin pain [5], whereas Morrissey et al. observed reduced activity in the adductor longus and gluteus medius muscle [6]. These findings suggest that patients with groin pain have decreased pelvic stability involving both the trunk and lower limbs. Previous studies have shown that the effects of groin pain originate from the pelvis and extend to the trunk. However, clinically examining the influence of the trunk and pelvis necessitates evaluating the muscles in the thorax and around the scapulae. In particular, the latissimus dorsi (LD) and trapezius muscles are affected by trunk rotation movements [7,8], making their assessment crucial. Until now, upper limb muscles influenced by the trunk, such as the LD and trapezius, have not been evaluated in groin pain research. Patients with groin pain showed significantly lower peak vertical GRF than healthy individuals in a continuous lateral hurdle hop task [9]. In addition, a study of individuals with a history of groin pain showed that vertical GRF tended to be higher during directional change tasks than in other tasks in individuals with a history of groin pain [10]. These studies have suggested that athletes change their movement strategies in some way due to the effects of groin pain, but the factors behind this have not yet been clarified. Furthermore, previous studies using EMG and GRF have been limited to patients with groin pain. Despite the high risk of recurrence in individuals with a history of groin pain, reports on its characteristics remain limited.

### 1.3. Muscle Synergy Analysis

Recently, muscle synergy analysis has been used to assess muscle coordination [11]. Muscle synergy refers to the concept of controlling multiple muscles as a functional group. Numerous studies have shown that the many muscle activations can be explained by a small number of muscle synergies or modules, and this approach has been widely applied in sport sciences [11,12,13,14] and orthopedics [15,16,17]. However, interpreting muscle activity data alone can be challenging. Therefore, in previous studies using muscle synergy analysis, biomechanical data and GRF data are often incorporated to better interpret the functions of the extracted synergies [12].

### 1.4. Current Gap

Despite the high recurrence rate in individuals with a history of groin pain, few studies have reported the physical characteristics of individuals with a history of groin pain. Although individuals with a history of groin pain have been shown to exhibit different strategic patterns from the thorax to the trunk and hip joints, no studies have visualized the coordination of muscles throughout the body. Furthermore, the relationship between muscle coordination and movement and parameters such as GRF, which explain muscle coordination, remains unclear.

### 1.5. Purpose and Hypotheses

Addressing these gaps, this pilot study combined muscle synergy analysis and GRF data to clarify differences in muscle coordination between individuals with a history of groin pain and healthy individuals. We hypothesized that individuals with a history of groin pain would show reduced contributions (weightings) of trunk and gluteal muscles in their muscle synergies. This hypothesis is based on previous findings showing reduced the internal oblique activation, transverse abdominis, gluteus medius, and adductor longus muscles in patients with groin pain, as muscle activity is reflected in muscle synergies. Although previous research has addressed the treatment and prevention of groin pain, few studies have focused on recurrence [10,18,19]. Understanding the muscle synergy characteristics of individuals with a history of groin pain may provide insights into the mechanisms underlying groin pain recurrence.

## 2. Materials and Methods

### 2.1. Participants

This study was designed as a cross-sectional pilot study to explore the feasibility of the methodology and to provide preliminary data regarding neuromuscular coordination patterns in athletes with and without a history of groin pain. Participants were allocated into groups intentionally based on their history of groin pain rather than by randomization. In this study, 15 athletes with a history of groin pain (GP) and 14 athletes with no history of groin pain (CON) (GP; age: 20.7 ± 1.3 years, CON; age: 20.6 ± 1.3 years, *p* = 0.62, d = 0.12, GP height: 164.5 ± 5.0 cm, CON height: 164.2 ± 8.8 cm, *p* = 0.33, d = 0.05, GP weight: 62.1 ± 8.0 kg, CON weight: 58.1 ± 8.8 kg, *p* = 0.09, d = 0.47, GP BMI: 22.9 ± 2.45, CON BMI: 21.6 ± 1.1 *p* = 0.05, d = 0.76 [mean ± standard deviation]) were recruited (Table 1 and Table 2). The athletes were male and female participants aged 18 to 30 years who competed in soccer, field hockey, and lacrosse. Previous studies have reported groin pain as a common injury in these sports [20,21,22]. These studies included members of teams affiliated with universities or adult sports clubs in Japan and participated in competitions at the amateur level. This study included only one non-elite athlete. Although his competitive experience and performance level were comparable to those of elite athletes, the inclusion of this single participant may limit the strict homogeneity of the sample. Nevertheless, given that the level of competition has been considered a stronger risk factor for groin pain [3] than training frequency, the potential influence of this athlete on the overall results is likely to be minimal. All participants reported their Copenhagen Hip and Groin Outcome Score (HAGOS) [23] to quantify hip dysfunction. Muscle activity data were successfully obtained from 9 and 11 participants in the GP and CON groups, respectively. GRF data was successfully obtained for all participants. The criteria for the GP group were as follows: (1) chronic groin pain lasting at least 2 weeks, (2) history of groin pain within the past 2 years, (3) groin pain defined by a healthcare specialist (doctors, athletic trainers, etc.), (4) absence of current orthopedic disease, and (5) absence of pain during cutting movements. CON was defined as athletes who met all the following criteria: (1) they currently had no other orthopedic conditions, (2) they experienced no groin pain, (3) they were able to perform cutting movements without discomfort, and (4) they had no history of chronic groin pain in the past 2 years. In patients with a history of groin pain, the leg supported during cutting was the leg that was most recently symptomatic. Groin pain was initially identified by either sports medicine physicians or certified athletic trainers; however, classification of pain type was performed only by physicians, as this required anatomical diagnosis based on clinical assessment (history, examination, and when indicated, imaging), while trainers could not reliably determine specific subtypes. The support legs for the CON group were chosen on the basis of which legs would be easiest for participants to use. After receiving a detailed explanation of the purpose, potential benefits, and risks associated with this study, participants provided written consent to participate. The Ethics Review Committee for Human Research approved this study (approval number: 2022-190).

### 2.2. Data Collection

**Figure 1:**
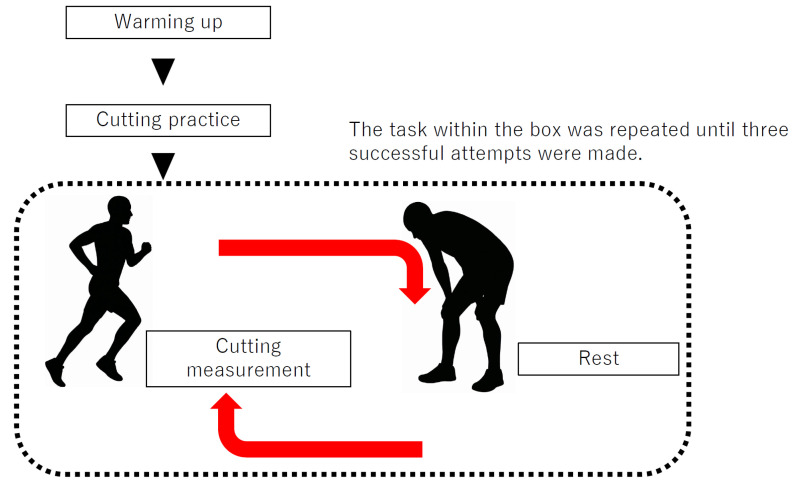
Experimental Procedure.

**Figure 2:**
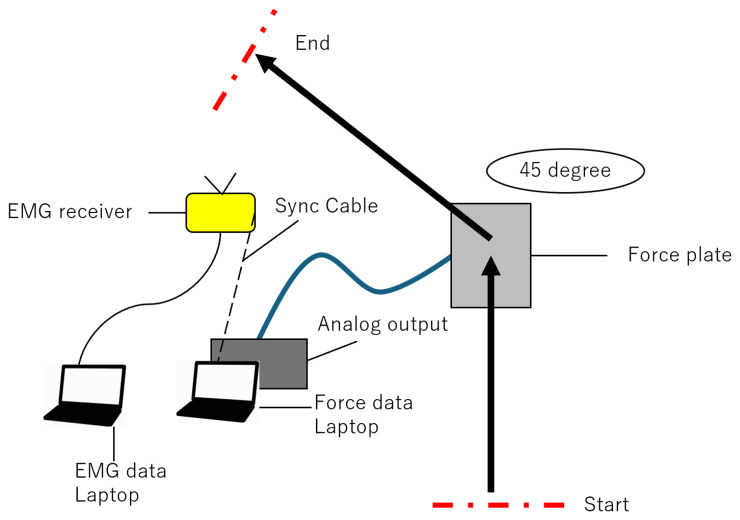
Experimental Environment for Cutting Measurements.

EMG data were collected from only 9 participants in the GP group and 11 participants in the CON group. Due to technical limitations associated with EMG acquisition in team-based testing reliable high-quality EMG signals could not be consistently obtained from all participants. Therefore, EMG measurements were successfully recorded only in these two groups. The selection of groups was not based on performance level, injury status, or any outcome-related factor, but solely on the feasibility of collecting valid EMG data during the testing sessions. The inclusion criteria for EMG measurements are the ability to acquire reliable, artifact-less signals during cutting measurements and the feasibility of electrode placement within the available test conditions. Participants for whom these criteria could not be met were excluded from EMG analysis. The participants warmed up (i.e., dynamic stretching, three practice changes in direction under controlled speed conditions, and three practice changes in direction at full effort), accelerated 5 m toward the force plate, and then performed a plant-and-cut maneuver at an angle of 45° starting from the center of the force plate. Participants were given enough rest time between each trial until all three trials were successfully completed to ensure adequate recovery and minimize the effects of fatigue (Figure 1). Output signals from the force plate embedded in the floor (Type 9286B, Kistler Instrument AG, Winterthur, Switzerland) were recorded synchronously along with EMG signals (Model DL-5000, S&ME Inc., Tokyo, Japan) (Figure 2). EMG signals and GRFs were measured at 1000 Hz.

### 2.3. GRF and EMG Procedure

**Figure 3:**
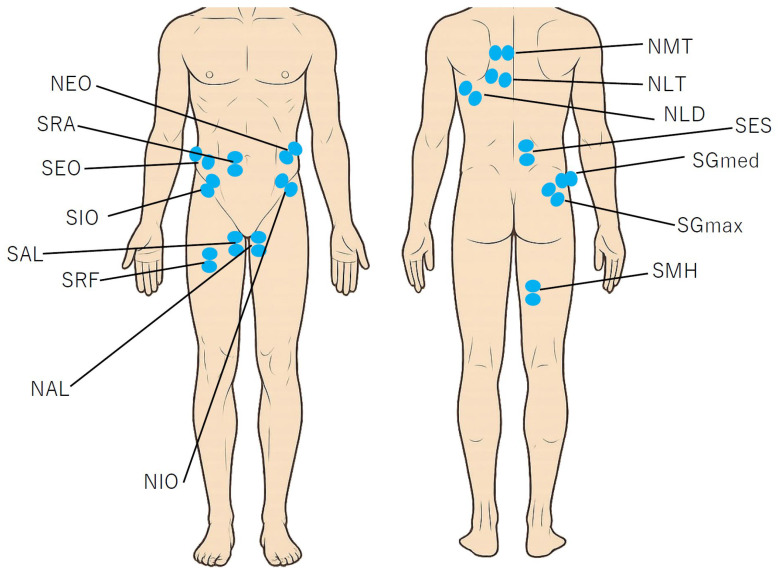
Sensor placement position during right leg measurement. SGmed, gluteus medius on the support leg; SRF, rectus femoris on the support leg; SMH, medial hamstring on the support leg; SIO, internal oblique on the support leg; NIO, internal oblique on the non-support leg; SEO, external oblique on the support leg; NEO, external oblique on the non-support leg; SAL, adductor longus on the support leg; NAL, adductor longus on the non-support leg; NLT, lower trapezius on the non-support leg; NMT, middle trapezius on the non-support leg; SES, erector spinae on the support leg; SRA, rectus abdominis on the support leg; NLD, latissimus dorsi on the non-support leg; SGmax, gluteus maximus on the support leg.

Force data were filtered using a fourth-order Butterworth filter with a cutoff frequency of 20 Hz. GRFs were calculated using MATLAB R2024a (MathWorks, Natick, MA, USA). All GRF data were normalized to body weight. Then, all data were interpolated to 101 points, and force data intervals were determined on the basis of the GRF data. Data collection began 100 msec before initial contact to consider the feedforward function of the trunk [24,25] and gluteus medius muscles [25]. In other words, 0% of the data represents 100 msec before initial contact. Support leg contact was defined as the point at which the force plate first exceeded 20 N (29% of the motion cycle), and support leg takeoff was defined as the point at which the vertical component of the force plate fell below 20 N (100% of the motion cycle) [26]. Registration occurred at the beginning of the acceleration phase (last positive forward GRF before the negative peak; 75% of the motion cycle). This process adjusts the start of the acceleration phase (75%) for all participants and accounts for differences in the braking/deceleration and acceleration phases between participants during the continuous waveform analysis [27]. The measured muscles were as follows (Figure 3): gluteus medius on the support leg (SGmed), rectus femoris on the support leg (SRF), medial hamstring on the support leg (SMH), internal oblique on the support leg (SIO), internal oblique on the non-support leg (NIO), external oblique on the support leg (SEO), external oblique on the non-support leg (NEO), adductor longus on the support leg (SAL), adductor longus on the non-support leg (NAL), lower trapezius on the non-support leg (NLT), middle trapezius on the non-support (NMT), erector spinae on the support leg (SES), rectus abdominis on the support leg (SRA), latissimus dorsi on the non-support leg (NLD), and gluteus maximus on the support leg (SGmax). Previous studies have pointed out that individuals with a history of GP have reduced thorax rotation compared with healthy individuals [10]. Based on previous studies, this study adopted NLT, NMT, SIO, NIO, SEO, and NEO as muscles related to thorax rotation and trunk rotation. Furthermore, previous studies have focused on the decrease in lateral pelvic tilt during single-leg squat [28] and drop landing [29]. Additionally, groin pain patients have reduced function of anterior pelvic tilt during active pelvic tilt movement [30]. SGmed, SAL, NAL, SRA, SES, and SGmax are muscles strongly associated with pelvic lateral tilt and anterior tilt function, so we focused on these muscles. The participants’ skin was shaved in the areas where the electrodes were to be placed and cleaned with alcohol-soaked cotton. Surface electromyography for noninvasive muscle assessment criteria was used as a guideline for electrode placement (http://seniam.org/). Disposable Ag/AgCl electrodes with a center-to-center spacing of 25 mm and a diameter of 10 mm were placed over each muscle. The application of the electromyography sensors was carefully performed by two physical therapists who palpated the muscles. All the data were time-interpolated over one cut to fit a normalized 1–101 points time base.

For each cut, EMG data was analyzed from 100 msec before support leg contact to support leg takeoff. The data were high-pass filtered (20 Hz, zero-lag fourth-order Butterworth filter), full-wave rectified, and low-pass filtered (10 Hz, zero-lag fourth-order Butterworth filter). Then, the EMG data were normalized using the peak activation of all muscle EMG values across all trials and interpolated over 101 points. The interpolated EMG data included the support leg contact, braking phase, and acceleration phase. An EMG matrix of 15 muscles × 101 data points ware combined for each trial (15 muscles × 101 data points × 3 trials) to extract synergies. The EMG matrix was normalized to the peak activation of each EMG signal during each cutting movement [31]. This EMG matrix was used to extract muscle synergies.

### 2.4. Extraction of Muscle Synergies

Muscle synergies were extracted using a non-negative matrix factorization (NMF) algorithm [32]. Each muscle synergy is represented as a time-invariant component that coactivates multiple muscles and is activated by a time waveform. NMF decomposes a matrix into two approximately non-negative matrices by minimizing the error between the original and reconstructed matrices. The two non-negative matrices correspond to synergy weighting and activation, respectively. The activation pattern of the muscles under condition (M) is expressed using Equation (1).

(1) $$M=\sum_{i=1}^{N}W_{i}C_{i}+\varepsilon \left(W_{i}\geq 0,C_{i}\leq 0\right)$$

where *N* is the number of synergies, *w_i_* is the weighting of a muscle in a muscle synergy *i*, *c_i_* denotes an activation that involves a relative contribution of the muscle synergy, and ***ε*** is the residual. The composition of the muscle synergy *w_i_* does not change within a given condition, whereas the activation *c_i_* does.

To determine the number of muscle synergies, the variability accounted for (VAF) was calculated on the basis of the dataset as a whole (*VAF global*) and each muscle VAF (*VAF local*) for muscle synergy (Equations (2) and (3)). *EMGo* is the original dataset of processed EMG signals, and *EMGr* is the reconstructed EMG matrix calculated using the NMF algorithm. The number of muscle synergies for each dataset was defined as the minimum number of synergies required to achieve a *VAF global* > 90% and *VAF local* > 75% [33].

(2) $$VAF\;global=\frac{{\left(EMGo-EMGr\right)}^{2}}{{EMGo}^{2}}\times 100$$

(3) $$VAF\;local=\frac{{{(EMGo}_{\left(m,i\right)}-{EMGrm}_{\left(m,i\right)})}^{2}\;\;\;\;\;\;}{{{EMGo}_{\left(m,i\right)}}^{2}}$$

The muscle synergies of all the participants were classified using MATLAB function (K-means). The number of clusters was set to three based on the number of synergies. Because the classification results depend on the initial values, the classification was repeated 100 times with different initial values, and the classification results with the smallest total distance between each point and the centroid of the cluster were adopted [34,35]. The similarity between muscle synergies was calculated from activation. The similarity was evaluated by scalar product (SP) [36]. Muscle synergy was considered significantly similar when the SP value 0.75 was <SP [37].

The weights of each muscle in muscle synergy were also compared in each group. The weight of the muscle synergy was calculated by determining the muscle contributing to each muscle synergy. Additionally, the weights of each muscle were compared between the groups.

### 2.5. Statistical Analysis

Since this was a pilot study, no prior power analysis was conducted. Therefore, the sample size was determined on the basis of the feasibility of recruitment and should be interpreted as exploratory. HAGOS, number of muscle synergies, and weightings of muscle synergies were verified for normality using the Shapiro–Wilk test. Independent *t*-tests or Mann–Whitney U tests were used to compare differences between groups. SPSS Statistics for Windows (version 29.0; IBM Corp., Armonk, NY, USA) was used for statistical analysis. The effect size was determined using Cohen d (small: >0.2; medium: >0.5; large: >0.8) [38]. In this study, 1d-SPM was used to compare the individual activity coefficients of muscle synergy, the anterior–posterior, lateral-medial, and vertical components of the force plate, and the composite vectors. After confirming normality using D’Agostini’s K-squared test, a 1d-SPM *t*-test 2 was performed [39]. In all tests, statistical significance was set at *p* < 0.05. MATLAB R2024a (MathWorks, Natick, MA, USA) was used for the statistical analysis of 1d-SPM (https://spm1d.org/). We analyzed time-series data using 1d-SPM. This approach enables hypothesis testing across the entire normalized waveform (e.g., joint angles, EMG signals) rather than at individual time points. By applying Random Field Theory, 1d-SPM controls for the multiple comparison problem inherent in high-dimensional signals and identifies clusters of consecutive time points where significant differences exist. This provides a straightforward way to evaluate group differences across continuous biomechanical data. To account for the imbalance in sex distribution between groups (GP: predominantly male; CON: predominantly female), we calculated effect size estimates (Hedges’ g) separately within each group to provide supplementary insight into potential sex-related differences. No formal statistical comparisons between sexes were performed due to the small subgroup sizes. The results of these sex-stratified effect size analyses are presented in the Supplementary Materials. Parameters showing large effect sizes were indicated based on effect size categories (small: >0.2; medium: >0.5; large: >0.8) [40].

## 3. Results

### 3.1. Participants

The HAGOSs were 90.1 and 100.0 for the GP and CON groups, respectively (Table 3). Minor functional limitations were found in the GP group (*p* = 0.001 d = 1.06). None of the athletes in the GP group reported groin pain during the execution of the cutting tasks. Therefore, the movement patterns observed in this group were not influenced by acute pain at the time of measurement.

### 3.2. Kinetic Date

**Figure 4:**
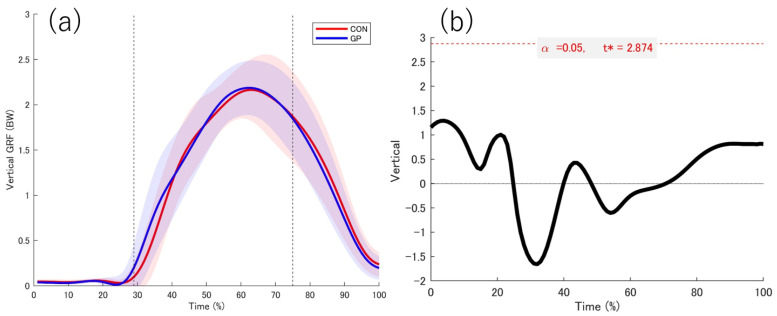
Vertical GRF. (**a**) The red and blue solid lines indicate the respective average values, and the shadows indicate the standard deviation. (**b**) The graph on the right shows the *t* value 1d-SPM. The significance probability was *p* < 0.05. The key finding of this figure is that no significant differences were observed in any interval between the two groups. GRF: Ground reaction force, t*: multiple-comparison corrected threshold.

**Figure 5:**
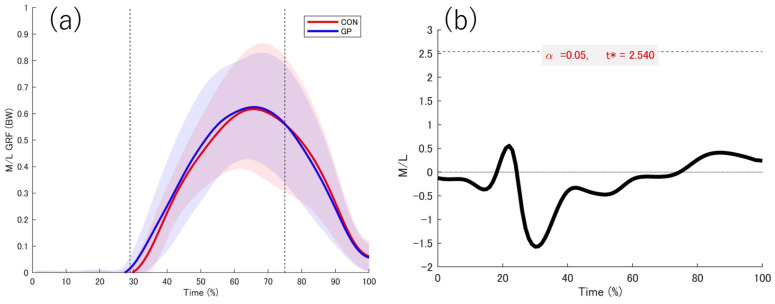
Medial-lateral directions GRF. (**a**) The red and blue solid lines indicate the respective average values, and the shadows indicate the standard deviation. (**b**) The graph on the far right shows the t value 1d-SPM. The significance probability was *p* < 0.05. The key finding of this figure is that no significant differences were observed in any interval between the two groups. GRF: Ground reaction force, t*: multiple-comparison corrected threshold.

**Figure 6:**
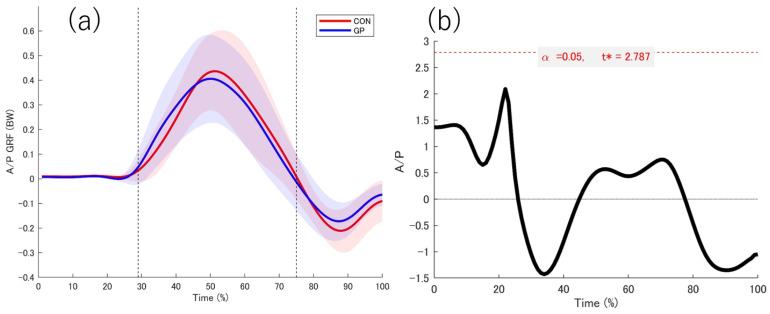
Anterior–posterior directions GRF. (**a**) The red and blue solid lines indicate the respective average values, and the shadows indicate the standard deviation. (**b**) The graph on the right shows the t value 1d-SPM. The significance probability was *p* < 0.05. The key finding of this figure is that no significant differences were observed in any interval between the two groups. GRF: Ground reaction force, t*: multiple-comparison corrected threshold.

**Figure 7:**
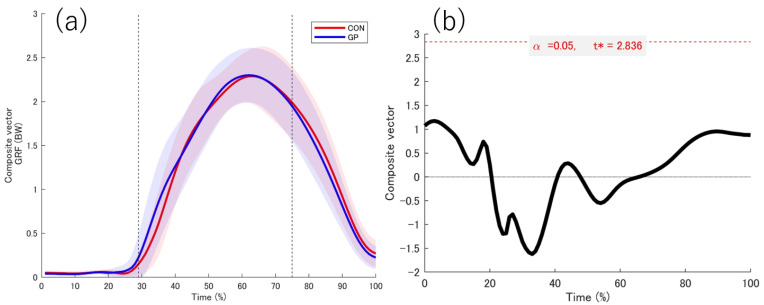
Composite vector from GRF. (**a**) The red and blue solid lines indicate the respective average values, and the shadows indicate the standard deviation. (**b**) The graph on the right shows the t value 1d-SPM. The significance probability was *p* < 0.05. The key finding of this figure is that no significant differences were observed in any interval between the two groups. GRF: Ground reaction force, t*: multiple-comparison corrected threshold.

The data obtained from the force plate while in contact with the support leg is shown (Figure 4, Figure 5, Figure 6 and Figure 7). No significant differences were observed between the groups in the force plate data for each component or composite component. The GRF data were normalized on the basis of the weight of the participant. Based on the vertical data of the GRF, the point at which the support leg contact first exceeds 20 N was defined as 29%. From the GRF data at the front and back, the point at which the backward and forward components switched was 75%. On the basis of these results, we defined the 0–28% phase as the pre-initial contact phase, 29% phase as the contact phase, 29–75% phase as the deceleration phase, and 76–100% phase as the acceleration phase. Additionally, Ex differences in vertical ground reaction forces across groups are shown in the Supplementary Materials (Figure S1).

### 3.3. Extracted Muscle Synergy

**Figure 8:**
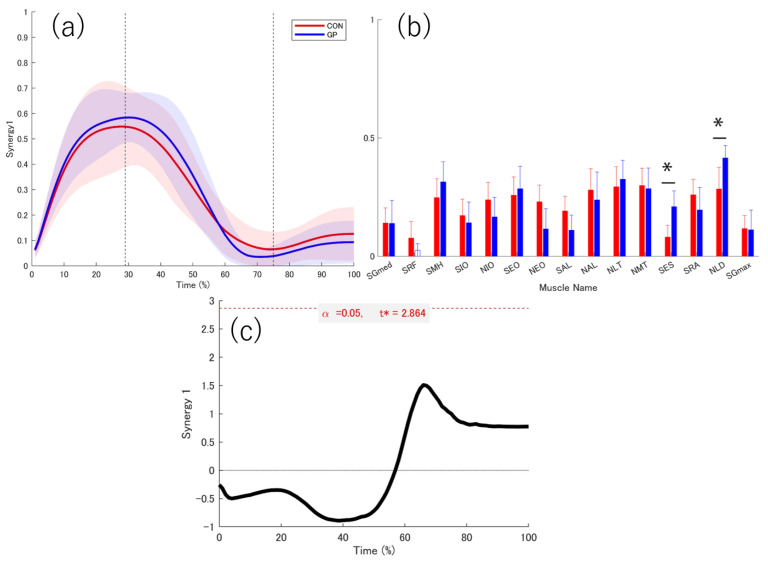
Weighting and activity coefficient of Synergy 1. Red indicates healthy individuals (CON), and blue indicates individuals with a history of groin pain (GP). The graph shows the mean values of (**a**) the activity coefficient and (**b**) the weighting of muscle synergy. The red and blue solid lines indicate the respective average values, and the shadows indicate the standard deviation. The red and blue bar graphs indicate the average values, and the bars indicate the standard deviation. The first dotted line at 29% represents the point of landing. The next dotted line at 75% represents the border between the deceleration phase and the acceleration phase. The 95% confidence interval of the average value of each muscle in the muscle synergy weighting was calculated, and the lower limit of the 95% confidence interval was taken as the lower limit of the 95% confidence interval. The muscles that do not contribute were indicated by white bar graphs. (**c**) The graph at the bottom center shows the statistical values of t in 1d-SPM. The significance probability was set at *p* < 0.05. The key finding in this graph is that Synergy 1 peaked during the landing phase. The muscle contributions of NES and NLD were significantly higher in GP. *: significant difference, SGmed, gluteus medius on the support leg; SRF, rectus femoris on the support leg; SMH, medial hamstring on the support leg; SIO, internal oblique on the support leg; NIO, internal oblique on the non-support leg; SEO, external oblique on the support leg; NEO, external oblique on the non-support leg; SAL, adductor longus on the support leg; NAL, adductor longus on the non-support leg; NLT, lower trapezius on the non-support leg; NMT, middle trapezius on the non-support leg; SES, erector spinae on the support leg; SRA, rectus abdominis on the support leg; NLD, latissimus dorsi on the non-support leg; SGmax, gluteus maximus on the support leg. t*: multiple-comparison corrected threshold.

**Figure 9:**
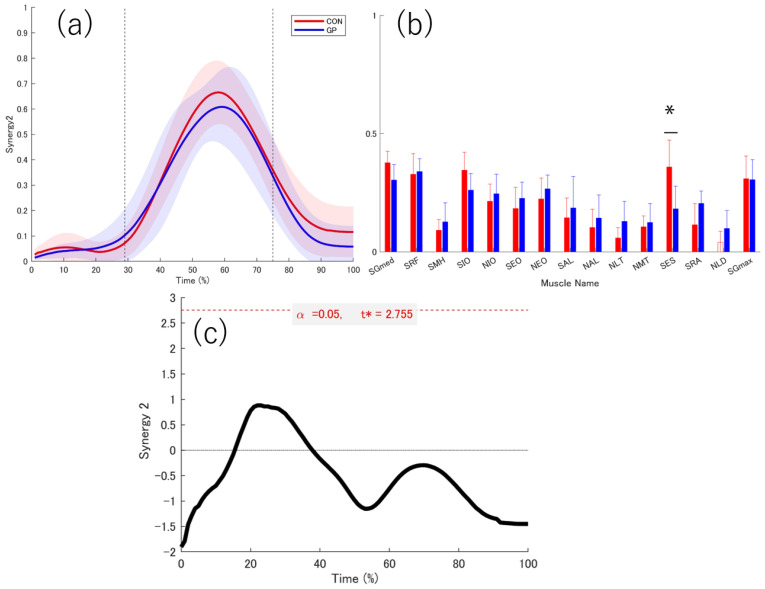
Weighting and activity coefficient of Synergy 2. Red indicates healthy individuals (CON), and blue indicates individuals with a history of groin pain (GP). The graph shows the mean values of (**a**) the activity coefficient and (**b**) the weighting of muscle synergy. The red and blue solid lines indicate the respective average values, and the shadows indicate the standard deviation. The red and blue bar graphs indicate the average values, and the bars indicate the standard deviation. The first dotted line at 29% represents the point of landing. The next dotted line at 75% represents the border between the deceleration phase and the acceleration phase. The 95% confidence interval of the average value of each muscle in the muscle synergy weighting was calculated, and the lower limit of the 95% confidence interval was taken as the lower limit of the 95% confidence interval. The muscles that do not contribute were indicated by white bar graphs. (**c**) The graph at the bottom center shows the statistical values of t in 1d-SPM. The significance probability was set at *p* < 0.05. The key finding in this graph is that Synergy 2 peaked during the deceleration phase. The muscle contribution of SES was significantly higher in CON. *: significant difference, SGmed, gluteus medius on the support leg; SRF, rectus femoris on the support leg; SMH, medial hamstring on the support leg; SIO, internal oblique on the support leg; NIO, internal oblique on the non-support leg; SEO, external oblique on the support leg; NEO, external oblique on the non-support leg; SAL, adductor longus on the support leg; NAL, adductor longus on the non-support leg; NLT, lower trapezius on the non-support leg; NMT, middle trapezius on the non-support leg; SES, erector spinae on the support leg; SRA, rectus abdominis on the support leg; NLD, latissimus dorsi on the non-support leg; SGmax, gluteus maximus on the support leg, t*: multiple-comparison corrected threshold.

**Figure 10:**
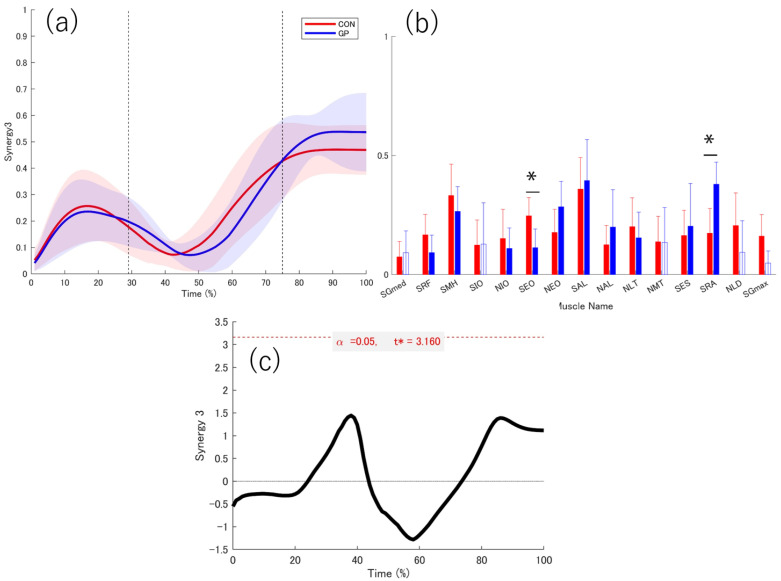
Weighting and activation coefficient of Synergy3. Red indicates healthy individuals (CON), and blue indicates individuals with a history of groin pain (GP). The graph shows the mean values of (**a**) the activation coefficient and (**b**) the weighting of muscle synergy. The red and blue solid lines indicate the respective average values, and the shadows indicate the standard deviation. The red and blue bar graphs indicate the average values, and the bars indicate the standard deviation. The first dotted line at 29% represents the point of landing. The next dotted line at 75% represents the border between the deceleration phase and the acceleration phase. The 95% confidence interval of the average value of each muscle in the muscle synergy weighting was calculated, and the lower limit of the 95% confidence interval was taken as the lower limit of the 95% confidence interval. The muscles that do not contribute were indicated by white bar graphs. (**c**) The graph at the bottom center shows the statistical values of t in 1d-SPM. The significance probability was set at *p* < 0.05. The key finding in this graph is that Synergy 3 peaked during the acceleration phase. The muscle contribution of SEO was significantly higher in CON, while the muscle contribution of SRA was significantly higher in GP. *: significant difference, SGmed, gluteus medius on the support leg; SRF, rectus femoris on the support leg; SMH, medial hamstring on the support leg; SIO, internal oblique on the support leg; NIO, internal oblique on the non-support leg; SEO, external oblique on the support leg; NEO, external oblique on the non-support leg; SAL, adductor longus on the support leg; NAL, adductor longus on the non-support leg; NLT, lower trapezius on the non-support leg; NMT, middle trapezius on the non-support leg; SES, erector spinae on the support leg; SRA, rectus abdominis on the support leg; NLD, latissimus dorsi on the non-support leg; SGmax, gluteus maximus on the support leg, t*: multiple-comparison corrected threshold.

There was no significant difference in the number of muscle synergies between the two groups (GP: 3.00 ± 0.47, CON: 3.45 ± 0.69, *p* = 0.114, d = 0.76). Three muscle synergies were extracted from both groups (Figure 8, Figure 9 and Figure 10).

### 3.4. Activation Coefficient

The peak values of each muscle synergy were calculated for Synergy 1 (GP: 28.22 ± 9.01%, CON: 31.00 ± 9.41%), Synergy 2 (GP: 58 ± 6.07%, CON: 58.50 ± 4.62%), and Synergy 3 (GP: 88.67 ± 12.29%, CON: 84.20 ± 12.55%). The similarities of muscle synergy were as follows: SP = 0.91 ± 0.04 for Synergy 1, SP = 0.91 ± 0.03 for Synergy 2, and SP = 0.91 ± 0.01 for Synergy 3. Thus, the three muscle synergies were significantly similar between the two groups (SP > 0.75). The activation coefficients for each muscle synergy were compared using 1d-SPM. In Synergies 1–3, no differences in the activation coefficients were observed between the groups in all sections.

### 3.5. Weightings

In Synergy 1, the SRF in the GP did not contribute significantly. The SES and NLD were significantly higher in the GP group than in the CON group (SES: *p* = 0.011, d = 1.257; NLD: *p* = 0.024, d = 0.945) (Figure 8). In Synergy 2, NLD did not contribute to synergy in the CON group. No significant contribution to SES was observed in CON (Figure 9). In Synergy 3, SGmed, SIO, NMT, NLD, and SGmax did not significantly contribute to GP. The SEO contribution was significantly higher in the CON group than in the GP group (*p* = 0.036, d = 1.137). In the GP group, the SRA contribution was significantly higher (*p* = 0.015, d = 1.353) than that in the CON group (Figure 10).

## 4. Discussion

This study aimed to use NMF and extract muscle synergies to investigate and compare neuromuscular control strategies between individuals with and without a history of groin pain during 45° cutting. This study is the first to investigate muscle synergy during 45° cutting in individuals with a history of groin pain. The results showed no difference in the dimensionality of the muscle synergies between the CON and GP groups. In both groups, all muscle synergies had three muscle synergies. The GP had significantly lower HAGOSs than the CON, even after returning to competitive sports. Additionally, GRFs during cutting in each group showed no changes; the movements being performed might be the same, but muscle synergy may differ in each group. These results revealed that understanding the functional dysfunction of groin pain in the GP might be possible from the characteristics of muscle synergy, not from the characteristics of movement. The results of this study partially supported our hypothesis. GP showed a decrease in muscle contribution, such as the SGmed and SIO muscles, compared to CON.

Importantly, no athletes in the GP group reported pain during testing. Thus, the altered movement strategies observed in this group are more likely to reflect long-term adaptations associated with a history of groin pain rather than immediate avoidance behaviour due to acute symptoms. While this strengthens the interpretation of chronic adaptations, it also limits the ability to examine movement responses under conditions of acute pain.

### 4.1. GRF

Although GRFs did not differ between groups, this finding supports prior evidence that global kinetic measures may lack sensitivity to detect subtle neuromuscular alterations in athletes with groin pain [41]. Instead, muscle synergy analysis revealed distinct coordination patterns that may provide a more sensitive marker of long-term adaptations.

### 4.2. Function of Muscle Synergy

The three muscle synergies extracted corresponded to distinct phases of the cutting movement and appeared to reflect specific neuromuscular control strategies. These interpretations are consistent with previous findings that muscle synergies represent modular control units associated with specific biomechanical functions, such as force generation in postural tasks [42], and that these synergies are preserved, merged, or fractionated depending on the integrity of motor cortical input [43]. Taken together, these findings suggest that the three synergies observed in this study form a temporal sequence of neuromuscular control strategies: from landing and stabilization (Synergy 1), through controlled deceleration (Synergy 2), to forward propulsion (Synergy 3). This sequence highlights the coordinated recruitment of muscle groups necessary for the effective and safe execution of the cutting maneuver.

### 4.3. Contact of the Support Leg

The muscle synergy most likely involved in the support leg contact phase was Synergy 1. The SRF did not contribute to the GP in the weighting of muscles in Synergy 1. SES and NLD contributions of the GP group were significantly higher than those of the CON group.

The SRF function stabilizes the support leg during single-leg landings [44]. The fact that the SRF did not contribute during landing in the GP group may be a factor that reduces stabilization of the supporting leg.

Furthermore, the higher muscle contributions of the SES and NLD may be related to the muscle connection. The NLD is anatomically connected to the SES through the lumbar fascia [45]. Previous studies have shown that the transmitted activation travels through the back to the buttocks and hip joints, causing hip external rotation in the prone position [46]. Therefore, although this study differs from previous studies in terms of position and movement, it is considered that the activation of muscle synergy is caused by muscle connections. External rotation of the hip joint by 30–50° during deep-squat positions has been reported to increase adductor muscle activation [47], which may in turn elevate groin loading. High adductor muscle activation increases the load on the groin and may be related to the recurrence of groin pain and symptoms [48]. However, since muscle activity was recorded from only one side, it was impossible to evaluate potential bilateral differences in activation.

### 4.4. Deceleration of the Support Leg

The muscle synergy most likely related to the deceleration of the supporting leg is Synergy 2, which did not contribute to the NLD weighting in the CON group. The SES contribution was significantly higher in the CON group than in the GP group.

The high contribution of the SES was characteristic of the CON group during the deceleration phase. erector spinae function is reportedly related to the active anterior tilt of the pelvis [49]. In this study, we were unable to collect pelvic kinematic data. However, the SES function may explain the problem of pelvis movement in the anterior tilt direction as a function of muscle synergy. In patients with groin pain, the range of motion of the anterior pelvic tilt decreases [30]. This functional decline affects function and joint angles in patients; however, individuals with a history of groin pain may have achieved joint angles using different strategies even though they had a functional decline.

The variations in NLD observed among participants may reflect several factors. Differences in training history and habitual upper limb usage could influence neuromuscular recruitment strategies. Hockey players exhibited muscle imbalances (hypertonicity/shortening) in the LD, suggesting potentially high loading on the upper limbs, particularly the scapular region [50]. Furthermore, lacrosse players exhibited greater upper limb muscle development compared to other overhead sports [51]. Such sport-specific habits may contribute to different activation patterns due to inter-individual differences in muscle morphology, such as fiber composition and attachment sites. Finally, normal inter-subject variation in movement control strategies is also considered a factor. Therefore, future studies should account for these factors when interpreting LD activation.

### 4.5. Acceleration Phase of the Support Leg

The muscle synergy most likely related to the acceleration of the supporting leg is Synergy 3. The weighting of Synergy 3 did not contribute to the weightings of the SGmed, SIO, NMT, NLD, and SGmax in the GP group. The SEO contribution was significantly higher in the CON group than in the GP group, and the SRA contribution was significantly higher in the GP group than in the CON group.

The peak value in Synergy 2 is related to acceleration but shows slight activation in the 29% or earlier section before landing. Minor activation before landing is clinically important in terms of the functions of the SGmed and SIO. The characteristics of patients with groin pain include a decrease in the function of the SGmed and SIO, which contribute to lateral stability of the pelvis [5,52,53]. The decreasing contribution from muscle synergy may have been a factor that reduced the pelvic stability. The inability to stably control the pelvis before landing may indicate that individuals with a history of groin pain are unable to avoid an increased risk to the affected leg during landing. However, it should be noticed that this study did not evaluate dynamic pelvic control using joint angles or other measures.

The results of Synergy 3 in this study showed a decrease in the muscle contribution of the gluteus maximus. The gluteus maximus is the primary muscle for hip extension [54]. A decrease in the muscle contribution of the gluteus maximus increases the load on other hip extensor muscles, such as the hamstrings, in the thigh [55]. In addition, muscle activation of the adductor longus reaches its maximum during the hip extension phase of a soccer kick. Therefore, the adductor longus is one of the muscles associated with hip extension [56]. A decrease in the muscle contribution of the gluteus maximus may lead to an increase in the contribution of the thigh muscles, which in turn may increase the load.

The presence or absence of the NMT and NLD muscle contributions may be affected by arm swinging during directional changes. Previous research has demonstrated that these muscles are engaged in trunk and thoracic rotation and serve as accessory stabilizers during multidirectional tasks, supporting efficient load transfer between the upper extremity and trunk [57,58]. However, in this study, the shoulder joint and muscles around the scapulae were not measured; therefore, these factors are not clear. Interestingly, athletes with a history of groin pain showed no contribution of the NLD and NMT to the extracted muscle synergies. Previous studies have reported that the NMT muscle in the LD assist in trunk rotation [8,59]. The lack of contribution observed in the GP group suggests that these athletes may be unable to supplement rotational control through upper body muscles, thereby increasing compensatory demands on the pelvis and hip joints. This divergence from established biomechanical roles of the LD and NMT indicates a potential maladaptive neuromuscular strategy associated with groin pain. Importantly, to our knowledge, this is the first study to identify reduced synergy contributions of trunk–shoulder muscles in athletes with a history of groin pain. This novel finding underscores the need to consider the integration of upper trunk muscles in both the assessment and rehabilitation of lumbopelvic dysfunction.

In a study that investigated the anatomical characteristics, a connection between the external oblique muscle and the adductor longus was reported. SEO was significantly lower in the GP group than in the CON group, suggesting that a decrease in muscle synergy contribution of the SEO of the support leg may increase the load on the adductor muscles. There is a muscle connection between the SEO and SAL [60], and muscle activation may be transmitted through muscle connections [61]. A decrease in the SEO during cutting may cause the impact to depend solely on the SAL, increasing the load on the SAL. A decrease in the muscle contribution of the SEO of the support leg in the GP group may have increased the risk of developing a decrease in adductor muscle contribution in the future. Furthermore, muscle contribution to SRA was significantly higher in the GP group. Excessive muscle synergy of the rectus abdominis during the acceleration phase may increase the stress on the pubic bone. The rectus abdominis muscle is attached to the pubic bone and antagonizes the adductor muscles to maintain the stability of the pubic bone [62]. An imbalance in the muscle contribution of the rectus abdominis and adductor muscles may increase the load on the groin, including the pubic bone, and may be associated with the recurrence of groin pain.

Clinically, these findings may indicate that strategies for preventing recurrence of groin pain should place emphasis on improving anterior pelvic tilt function, enhancing preparatory activation of the gluteal and trunk muscles prior to support-leg contact, and facilitating upper limb muscle activation during trunk rotation. Such interventions could potentially help to reduce repetitive loading on the groin region and thereby lower the likelihood of symptom recurrence. Accordingly, recurrence prevention programs might consider targeting anterior pelvic tilt function (e.g., cat-and-dog exercise), preparatory gluteal and trunk muscle activation before support-leg contact (e.g., hip hitch exercise), and upper limb muscle activation during trunk rotation (e.g., world’s greatest exercise) to address reduced muscle contributions. Targeted strategies of this kind may contribute to improved trunk–pelvic control and a decreased risk of recurrence.

### 4.6. Limitations

There are some limitations to this study. The sample size was small, limiting generalizability. A key limitation of this study is the imbalance in sex distribution between groups (GP: predominantly male; CON: predominantly female). Effect size estimates stratified by sex were provided as supplementary analyses; however, the small subgroup sizes prevent firm conclusions. Future studies with larger and more balanced samples are needed to clarify potential sex-related influences. This study only included healthy young athletes at the amateur level, limiting its generalizability. Future research should investigate athletes with a range of training experiences.

Groin pain was evaluated by various healthcare professionals, and different assessment methods were used due to the lack of standardized assessment scales. This study recruited patients presenting various types of groin pain. Therefore, it was not possible to focus on specific types of groin pain and clarify their characteristics. The variability in the classification of groin pain may have influenced biomechanical characteristics and muscle activation strategies. However, previous studies have shown that the same treatment protocol was used for all types of groin pain and that patients were able to return to sports [63,64,65]. Therefore, even if the type of the injury is the same, there may be different characteristics depending on the type of the injury, and there may be common characteristics regardless of the type of the injury. Biomechanical indicators such as joint angles could not be obtained in this study. As a result, it was not possible to clarify the reasons for muscle synergy based on movement indicators.

This study has two main limitations regarding muscle activity. First, because one side upper limb muscle activity was not measured, potential bilateral differences may have been overlooked, limiting generalizability. Second, because static contractions were not employed, comparability with studies using controlled isometric protocols is limited. Future research should address these issues to enhance the interpretation and application of findings.

Furthermore, a history of groin pain is recognized as a risk factor for recurrence, but it remains unclear whether the observed neuromuscular differences are a result of previous injuries or indicative of underlying factors. Therefore, the results of this study should be interpreted with caution. Further prospective studies are needed to determine whether muscle synergy patterns can serve as an early indicator of groin pain risk.

## 5. Conclusions

In conclusion, although the number of muscle synergies was similar between athletes with and without a history of groin pain, their composition differed. These findings indicate that athletes with history of groin pain adopt distinct neuromuscular coordination strategies during cutting maneuvers, particularly in phases requiring impact absorption and change of direction. Such long-term adaptations may contribute to the risk of recurrent groin problems and should be considered in the development of preventive interventions.

## Figures and Tables

**Table 1:** Basic information and injury history of the GP group.

No.	Sex	Age	Height (cm)	Weight (kg)	Level of Athletic Activity	Type of Sports	Competitive Level	Dominant Limb	Injured Limb	Types of Groin Pain
1	M	20	169.5	60.5	Elite	Soccer	High	R	R	Adductor
2	M	22	169.5	81.9	Elite	Hockey	High	R	R	Iliopsoas
3	M	21	173.2	61.2	Elite	Soccer	High	L	L	Adductor
4	M	20	164.0	50.0	Elite	Hockey	High	R	R	N/A *
5	M	20	163.0	66.0	Elite	Lacrosse	High	R	R	Hip
6	M	22	166.8	61.0	Non-Elite	Hockey	High	R	L	Pubic
7	M	21	162.0	55.7	Elite	Soccer	Middle	R	R	Adductor
8	M	22	169.4	76.0	Elite	Lacrosse	High	R	R	Iliopsoas
9	M	21	165.0	62.4	Elite	Soccer	Middle	R	R	Adductor
10	M	19	160.9	56.7	Elite	Soccer	Middle	L	L	Adductor
11	F	18	161.5	59.8	Elite	Soccer	High	R	L	Pubic
12	F	20	155.7	57.6	Elite	Hockey	High	R	L	Hip
13	M	21	169.4	63.2	Elite	Hockey	High	R	R	Hip
14	M	23	161.5	63.0	Elite	Hockey	High	R	R	Adductor
15	F	21	156.8	56.0	Elite	Hockey	High	R	L	Hip

Elite: ≧5 times per week, Non-Elite: ≦5 times per week, High: Players belonging to teams in Japan’s first division, Middle: Players belonging to teams in Japan’s second division or lower leagues, N/A *: For one athlete, the anatomical subtype of groin pain could not be confirmed because the assessment was performed by a trainer rather than a physician.

**Table 2:** Basic information about CON Group.

No.	Sex	Age	Height (cm)	Weight (kg)	Level of Athletic Activity	Type of Sports	Competitive Level	Dominant Limb
1	F	19	155.0	49.5	Elite	Hockey	High	R
2	F	22	160.9	55.9	Elite	Lacrosse	High	L
3	F	20	162.5	52.0	Elite	Hockey	High	R
4	F	19	160.6	51.6	Elite	Soccer	High	R
5	M	20	170.5	66.5	Elite	Hockey	High	L
6	M	20	181.8	76.0	Elite	Soccer	Middle	R
7	F	22	159.5	52.8	Elite	Lacrosse	High	R
8	F	22	164.0	61.4	Elite	Soccer	High	R
9	F	20	156.5	52.0	Elite	Hockey	High	R
10	M	22	182.4	76.2	Elite	Soccer	High	R
11	F	20	168.0	57.0	Elite	Soccer	High	R
12	M	23	163.7	57.1	Elite	Hockey	High	R
13	F	20	158.2	54.3	Elite	Soccer	High	R
14	F	19	155.0	51.6	Elite	Hockey	High	R

Elite: ≧5 times per week, High: Players who have played for teams in Japan’s first division, Middle: Players who have played in Japan’s second division or lower leagues.

**Table 3:** Scores for each group in HAGOS.

HAGOS (%)	GP		CON		*p* Value	Effect Size d
HAGOS	90.1	57.7–100.0	100.0	85.7–100.0	0.001	1.06
Symptoms Score	85.7	64.3–100.0	100.0	95.0–100.0	<0.001	1.51
Pain Score	95.0	67.5–100.0	100.0	90.0–100.0	0.002	1.12
Physical Function, Daily Living Score	100.0	90.0–100.0	100.0	100.0–100.0	0.07	1.00
Function, Sports, and Recreational Activities Score	96.9	65.6–100	100.0	93.7–100.0	0.02	1.00
Participation in Physical Activities Score	100.0	12.5–100.0	100.0	100.0–100.0	0.07	0.70
Quality of Life Score	90.0	40.0–100.0	100.0	80.0–100.0	0.005	1.06

The median and range value for each group are shown. The results of the Shapiro–Wilk test showed that all data followed a non-normal distribution.

## Data Availability

The author will share data upon request from readers.

## Supplementary Materials

The following supporting information can be downloaded at: https://www.mdpi.com/article/10.3390/sports13100338/s1, Figure S1: Sex differences in vertical ground reaction forces across groups; Figure S2: Sex differences in medial–lateral ground reaction forces across groups; Figure S3: Sex differences in anterior–posterior ground reaction forces across groups; Figure S4: Sex differences in composite vector ground reaction forces across groups; Figure S5: Differences between males, females, and groups in Synergy 1; Figure S6: Differences between males, females, and groups in Synergy 2; Figure S7: Differences between males, females, and groups in Synergy 3.

## Abbreviations

The following abbreviations are used in this manuscript:

GRF	Ground reaction force
GP	Group of with a history of groin pain
CON	Control
SGmed	Support leg gluteus medius
SRF	Support leg rectus femoris
SMH	Support leg medial hamstring
SIO	Support leg internal oblique
NIO	Non-support leg internal oblique
SEO	Support leg external oblique
NEO	Non-support leg external oblique
SAL	Support leg adductor longus
NAL	Non-support leg adductor longus
NLT	Non-support leg lower trapezius
NMT	Non-support leg middle trapezius
SES	Support leg erector spinae
SRA	Support leg rectus abdominis
NLD	Non-support leg latissimus dorsi
SGmax	Support leg gluteus maximus
EMG	Electromyography
HAGOS	Copenhagen Hip and Groin Outcome Score
