# Supplementary material for: Muscle Synergy During Cutting Movements in Athletes with a History of Groin Pain
Source: Sports (Basel). 2025 Oct 2;13(10):338. doi: 10.3390/sports13100338 (PMC12567835; doi:10.3390/sports13100338)
Supplement: Supplementary file 1 [file sports-13-00338-s001.zip › sports-3844813-supplementary.pdf]

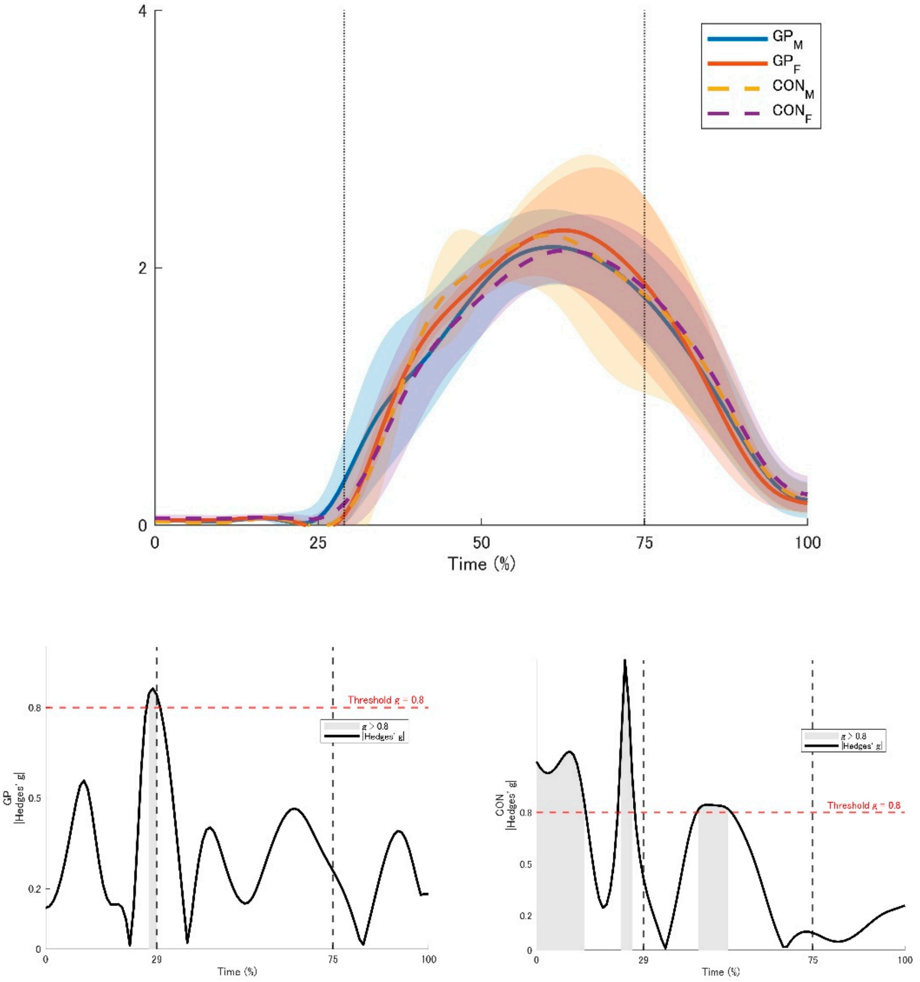

Figure S1. Sex differences in vertical ground reaction forces across groups.

The top panel shows group-mean values with standard deviations, separated by sex within each group. The lower panels illustrate Hedge's g: the left panel presents sex differences within the GP group, and the right panel presents sex differences within the CON group.

Abbreviations: GP_M, males with a history of groin pain; GP_F, females with a history of groin pain; CON_M, healthy males; CON_F, healthy females.

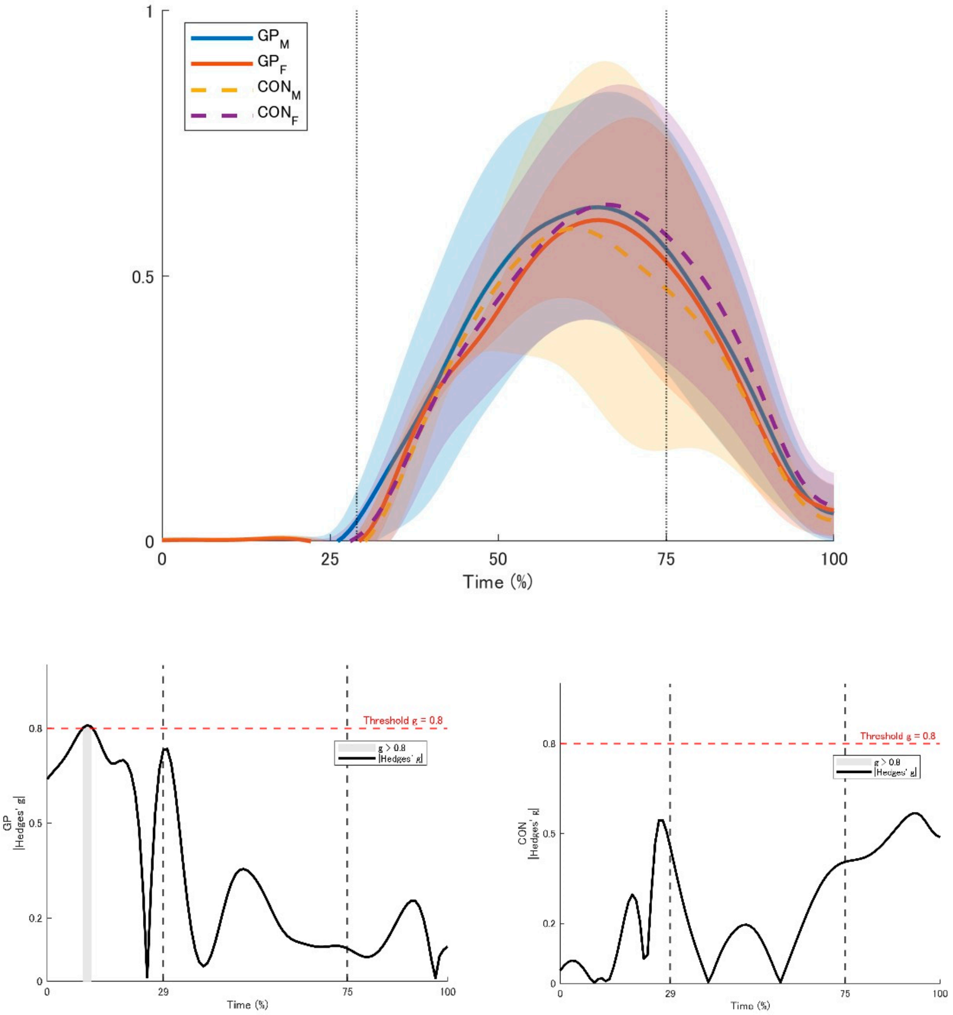

Figure S2. Sex differences in medial–lateral ground reaction forces across groups

The top panel shows group-mean values with standard deviations, separated by sex within each group. The lower panels illustrate Hedge's g: the left panel presents sex differences within the GP group, and the right panel presents sex differences within the CON group.

Abbreviations: GP_M, males with a history of groin pain; GP_F, females with a history of groin pain; CON_M, healthy males; CON_F, healthy females.

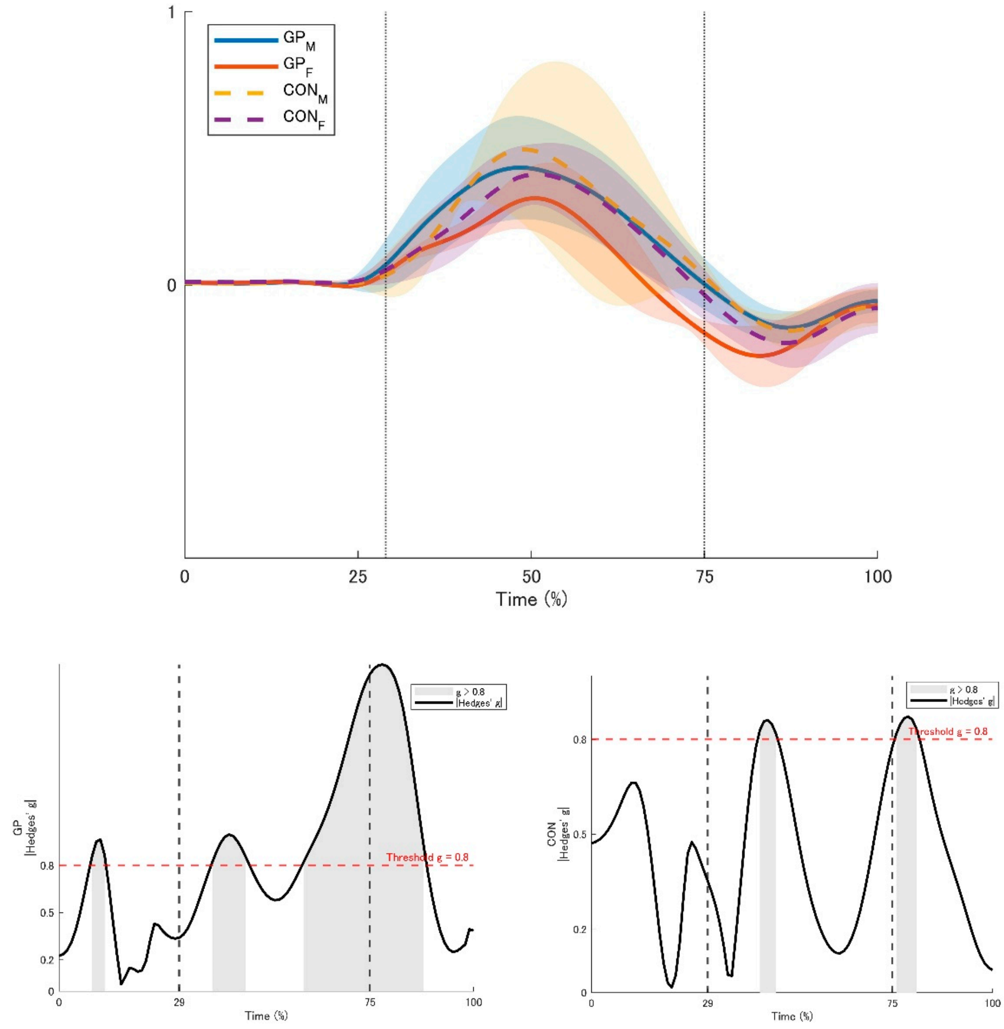

Figure S3. Sex differences in anterior–posterior ground reaction forces across groups

The top panel shows group-mean values with standard deviations, separated by sex within each group. The lower panels illustrate Hedge's g: the left panel presents sex differences within the GP group, and the right panel presents sex differences within the CON group.

Abbreviations: GP_M, males with a history of groin pain; GP_F, females with a history of groin pain; CON_M, healthy males; CON_F, healthy females.

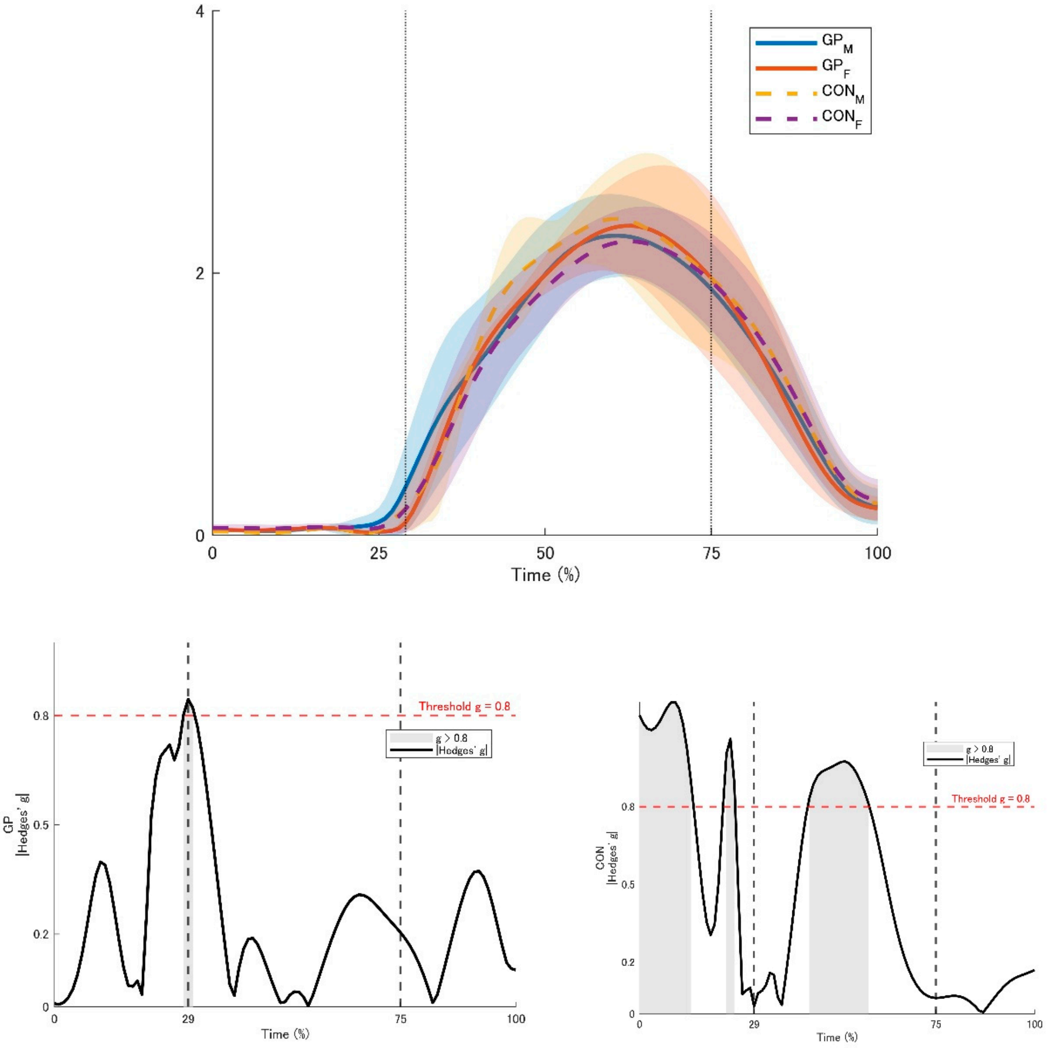

Figure S4. Sex differences in composite vector ground reaction forces across groups

The top panel shows group-mean values with standard deviations, separated by sex within each group. The lower panels illustrate Hedge's g: the left panel presents sex differences within the GP group, and the right panel presents sex differences within the CON group.

Abbreviations: GP_M, males with a history of groin pain; GP_F, females with a history of groin pain; CON_M, healthy males; CON_F, healthy females.

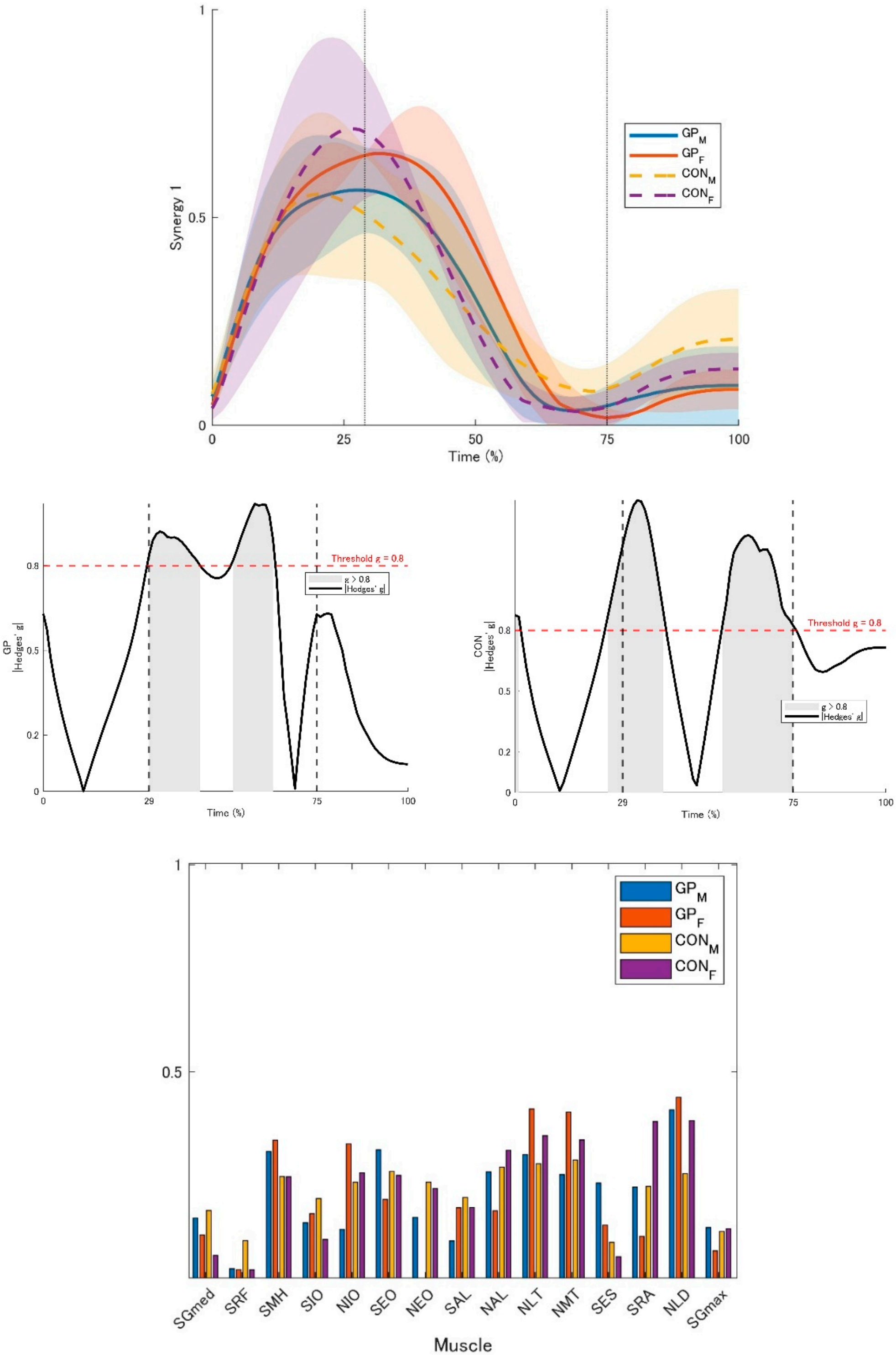

Figure S5. Differences between males, females, and groups in Synergy 1

The top panel shows the activation coefficients of muscle synergies classified by sex within each group, along with their mean and standard deviation. The middle panel displays the Hedge's g for the activation coefficients: the left panel shows sex differences within the GP group, and the right panel shows sex differences within the CON group. The bottom bar graph shows the weighting of muscle synergies classified by sex within each group, with the mean

explicitly indicated. The 29% dotted line indicates landing, while the 75% dotted line marks the transition between deceleration and acceleration. The red dotted line indicates the threshold for large effect size (g > 0.8).

Abbreviations: GP_M, males with a history of groin pain; GP_F, females with a history of groin pain; CON_M, healthy males; CON_F, healthy females.

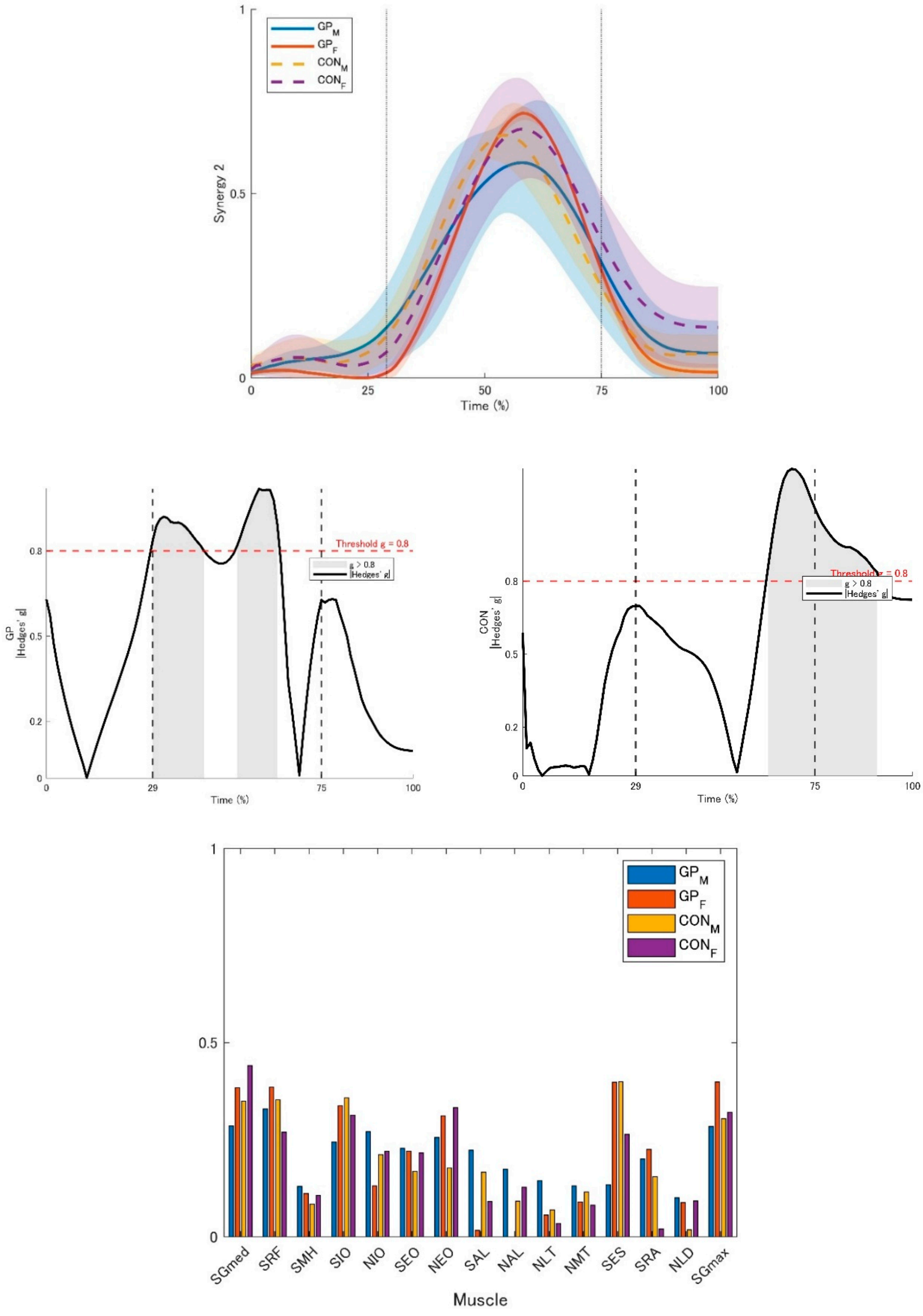

Figure S6. Differences between males, females, and groups in Synergy 2

The top panel shows the activation coefficients of muscle synergies classified by sex within each group, along with their mean and standard deviation. The middle panel displays the Hedge's g for the activation coefficients: the left panel shows sex differences within the GP group, and the right panel shows sex differences within the CON group. The bottom bar graph shows the weighting of muscle synergies classified by sex within each group, with the mean explicitly indicated. The 29% dotted line indicates landing, while the 75% dotted line marks the transition between deceleration and acceleration. The red dotted line indicates the threshold for large effect size (g > 0.8).

Abbreviations: GP_M, males with a history of groin pain; GP_F, females with a history of groin pain; CON_M, healthy males; CON_F, healthy females.

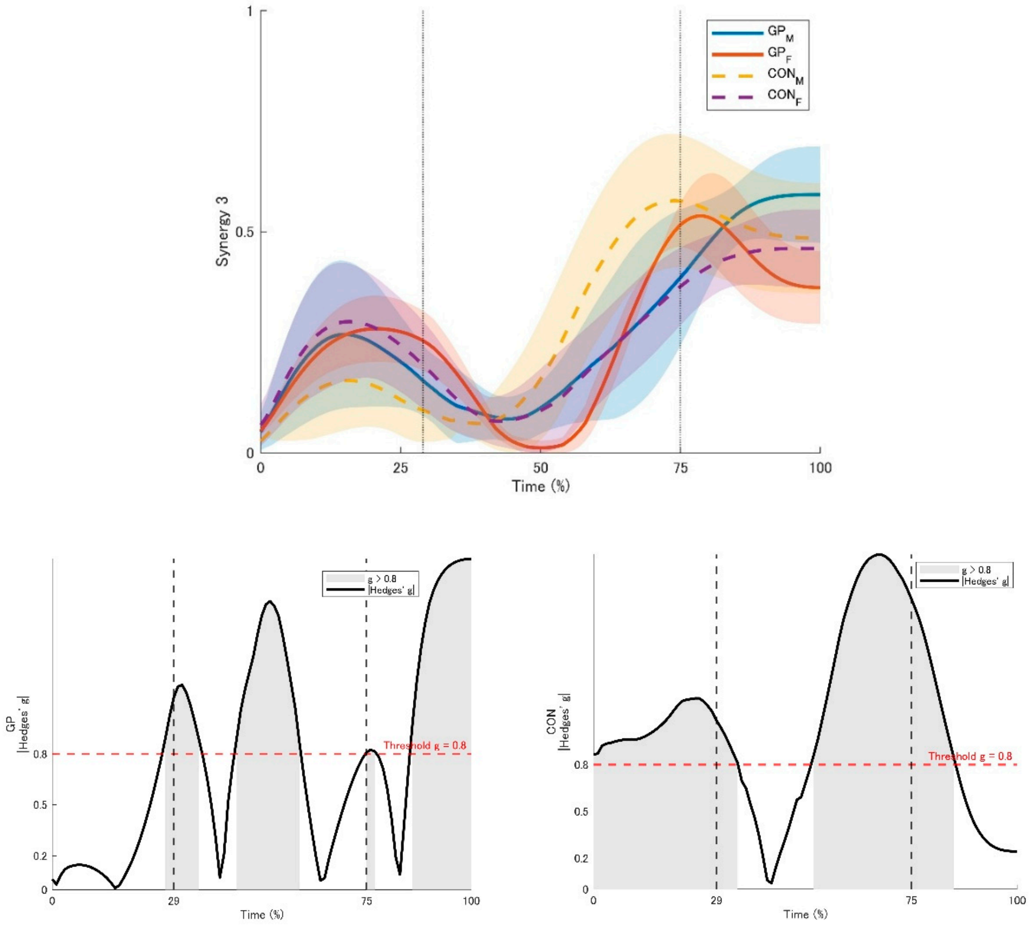

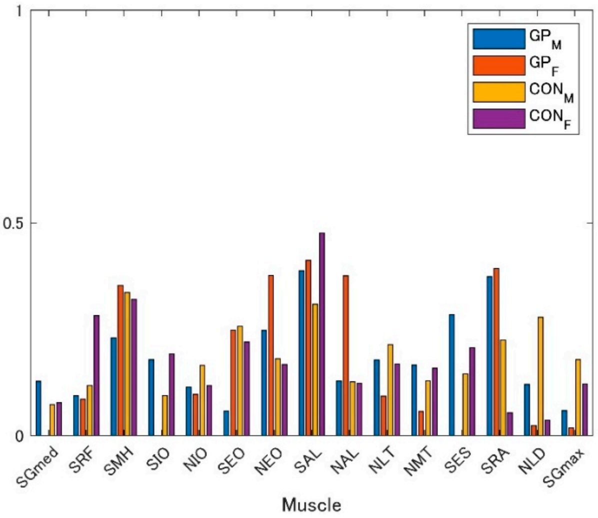

Figure S7. Differences between males, females, and groups in Synergy 3

The top panel shows the activation coefficients of muscle synergies classified by sex within each group, along with their mean and standard deviation. The middle panel displays the Hedge's g for the activation coefficients: the left panel shows sex differences within the GP group, and the right panel shows sex differences within the CON group. The bottom bar graph shows the weighting of muscle synergies classified by sex within each group, with the mean explicitly indicated. The 29% dotted line indicates landing, while the 75% dotted line marks the transition between deceleration and acceleration. The red dotted line indicates the threshold for large effect size ($g > 0.8$).

Abbreviations: GP_M, males with a history of groin pain; GP_F, females with a history of groin pain; CON_M, healthy males; CON_F, healthy females.

Results

Ground Reaction Forces (GRF)

In the GP group, females exhibited greater vertical GRF between 27–29% of the stance phase. In the CON group, females showed higher values between 0–13% and 23–26%, whereas males demonstrated higher values between 44–52% (Figure S1).

For mediolateral GRF, females in the GP group were higher between 9–11%, while no sex-related differences were observed in the CON group (Figure S2).

For anteroposterior GRF, females in the GP group showed higher values between 8–11%, 37–45%, and 59–88%. In the CON group, males were higher between 42–46%, 76–77%, and 79–81%, whereas females were higher at 78% (Figure S3).

For the composite vector, males in the GP group were higher between 28–30%, while in the CON group, females were higher between 0–13%, 22–24%, 52%, and 53–58%, and males were higher between 43–50% and at 51% (Figure S4).

Activation Coefficients of Muscle Synergy

Synergy 1:

In the GP group, females showed higher activation in Synergy 1 between 29–43% and 52–63%, whereas in the CON group, females were higher at 0–1% and 56–75%, and males were higher between 25–40% (Figure S5).

Synergy 2:

For Synergy 2, males in the GP group exhibited higher values between 8–34%, whereas females were higher between 53–59%. In the CON group, females were higher between 63–91% (Figure S6).

Synergy 3:

For Synergy 3, females in the GP group were higher between 27–36%, while males were higher between 44–59% and 86–100%. In the CON group, females were higher between 0–34% and 75–77%, whereas males were higher between 52–85% (Figure S7).

Weightings of Muscle Synergy

- **Synergy 1:**
  In the GP group, females exhibited higher weightings in NIO (g = 1.939), NLT (g = 0.810), and NMT (g = 1.084), whereas males showed higher values in NEO (g = 1.107) and SES (g = 0.927). In the CON group, males demonstrated higher weightings in SGmed (g = 0.901), while females were higher in SRA (g = 1.400) (Figure S5).
- **Synergy 2:**
  In the GP group, females showed higher values in SGmed (g = 0.818) and SES (g = 1.875), whereas males exhibited higher weightings in NIO (g = 0.945), SAL (g = 0.849), and NAL (g = 1.011). In the CON group, females demonstrated higher weightings in SGmed (g = 1.220) and NEO (g = 1.099), while males were higher in SRA (g = 0.893). NLD also tended to be higher in females (g = 0.939) (Figure S6).
- **Synergy 3:**
  In the GP group, males showed higher weightings in SGmed (g = 0.922) and tended to be higher in SES (g = 1.097), whereas females exhibited markedly higher values in SEO (g = 2.832). In the CON group, females demonstrated higher values in SRF (g = 1.241), while males were higher in SRA (g = 0.991). NLD also tended to be higher in males (g = 1.099) (Figure S7).
